# Left paraduodenal hernia treated by single-incision laparoscopic surgery: a case report

**DOI:** 10.1186/s40792-021-01292-7

**Published:** 2021-09-21

**Authors:** Noboru Hasegawa, Hiroshi Takeyama, Yozo Suzuki, Shingo Noura, Kazuki Odagiri, Yoshitomo Yanagimoto, Masafumi Yamashita, Junzo Shimizu, Tomono Kawase, Hiroshi Imamura, Takashi Iwazawa, Naohiro Tomita, Keizo Dono

**Affiliations:** Department of Surgery, Toyonaka Municipal Hospital, 4-14-1 Shibahara-cho, Toyonaka, Osaka 560-8565 Japan

**Keywords:** Internal hernia, Left paraduodenal hernia, Single-incision laparoscopic surgery (SILS)

## Abstract

**Background:**

Paraduodenal hernia is a rare internal hernia which accounts for only 1% of all intestinal hernias. There have been limited reported cases of paraduodenal hernia treated by laparoscopic surgery. We report a case of left paraduodenal hernia that was successfully treated by single-incision laparoscopic surgery (SILS).

**Case presentation:**

A 17-year-old woman presented with left upper abdominal pain. An abdominal enhanced multi-detector computed tomography demonstrated encapsulated cluster of small bowel loops in the left upper quadrant which passed through the dorsal side of the inferior mesenteric vein, and showed that blood flow of the prolapsed small bowel was preserved. We preoperatively diagnosed left paraduodenal hernia without ischemia or necrosis. We performed elective SILS because she was a young actress training school student and cosmetic benefit was thought to be important. We pulled out the protruded small bowel and closed a defect with a running suture by SILS. The patient was discharged 3 days after the surgery with no complications.

**Conclusions:**

We reported the case of left paraduodenal hernia successfully diagnosed and treated by SILS.

## Background

Internal hernia is defined as the protrusion of a viscus through a normal or abnormal peritoneal or mesenteric defect within the confines of the peritoneal cavity [1]. Internal hernia is rare and accounts for only 1% of all intestinal hernias [1]. Paraduodenal hernia is most popular internal hernia and accounts for approximately half of internal hernias [1]. However, there have been limited reported cases of paraduodenal hernia treated by laparoscopic surgery and there has been no reported case of paraduodenal hernia treated by single-incision laparoscopic surgery (SILS) [2]. Here, we report a case of left paraduodenal hernia that was successfully treated by SILS.

## Case presentation

**Fig. 1:**
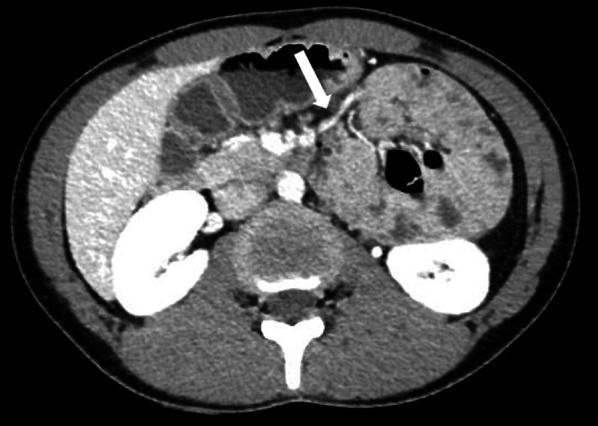
A multi-detector contrast-enhanced computed tomography scan showed no signs of ischemic findings. The small bowel was congregated on the left upper quadrant of the abdomen through the dorsal side of the inferior mesenteric vein (white arrow)

The patient was a 17-year-old woman who presented with left abdominal pain and was admitted to our hospital. She had repeatedly presented with sudden abdominal pains since childhood, but she had been diagnosed with constipation without a detailed examination such as computed tomography (CT). An abdominal enhanced multi-detector CT demonstrated encapsulated cluster of small bowel loops in the left upper quadrant which passed through the dorsal side of the inferior mesenteric vein (IMV), and showed that blood flow of the prolapsed small bowel was preserved (Fig. 1). Laboratory findings showed slightly elevated inflammation (WBC: 9100/μl, CRP: 0.02 mg/dl, Lactate: 0.70 mmol/L). We preoperatively diagnosed left paraduodenal hernia without ischemia or necrosis. Also we had concerned congenital factor and we carefully assessed CT with experienced radiologists. CT findings did not reveal any congenital factors, such as abnormal bowel rotation and revealed only a left paraduodenal hernia as an abnormal finding. We did not select emergency surgery since there were no signs of necrotic bowel in physical examination, blood test and CT findings. Four days after admission to our hospital, we performed elective SILS, because she was a young actress training school student and cosmetic benefit was thought to be important.

**Fig. 2:**
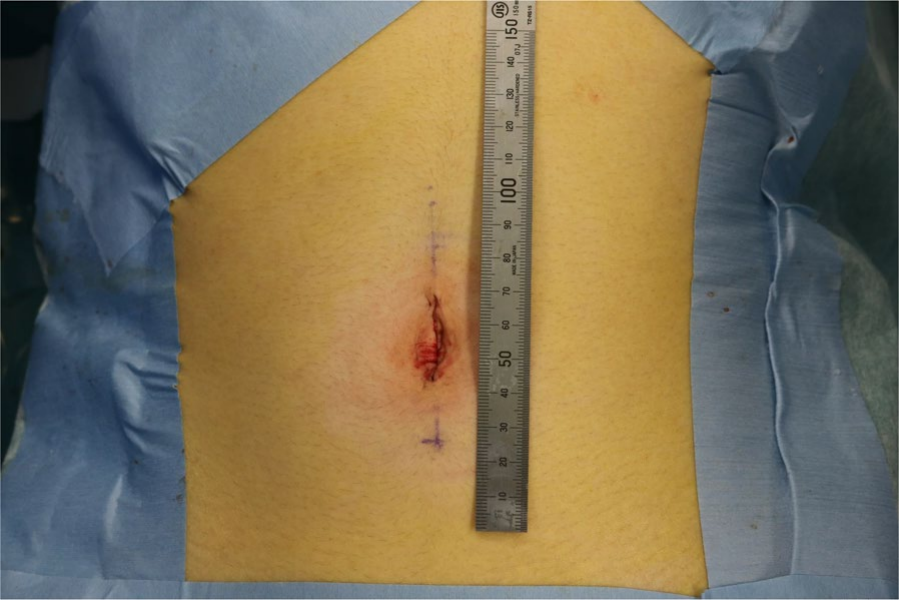
Postoperative scar after single-incision laparoscopic surgery. The diameter of the incision was 2.5 cm

**Fig. 3:**
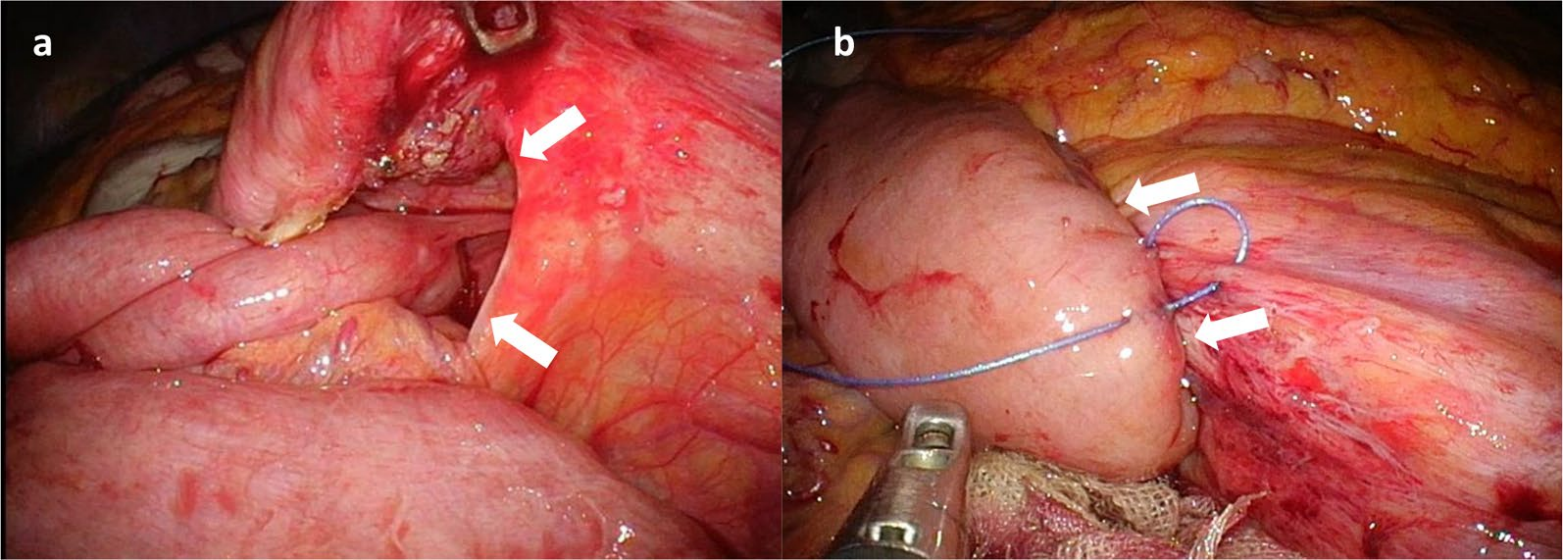
Images during surgery. **a** Landzert’s fossa (white arrow) was revealed after the reduction of the hernia contents. **b** Closing of the defect (white arrow) by suture

We made a 2.5-cm vertical single incision at the umbilicus (Fig. 2) and applied a sealing device (LAP-PROTECTOR™ Mini-type; Hakko, Nagano, Japan) to the wound. We used a single-port access device (E∙Z Access; Hakko) with inserted three 5-mm trocars (to accommodate a 5-mm flexible scope and surgical devices). Exploratory laparoscopy identified the protruded small bowel through the hernia orifice and we confirmed the definitive diagnosis as left paraduodenal hernia. First, we pulled out the protruded small bowel and the orifice was clarified (Fig. 3a). Next, we closed the defect with suture using a nonabsorbable yarn (V-Loc™ PBT; Medtronic, Minneapolis, USA) (Fig. 3b). The patient was discharged 3 days after the surgery with no complications.

## Discussion

Paraduodenal hernia is classified into two types: right paraduodenal hernia, in which the intestinal tract prolapses to the right side through the dorsal side of the superior mesenteric vein (Waldeyer’s fossa); and left paraduodenal hernia, in which the intestinal tract prolapses to the dorsal side of the descending mesocolon through the dorsal side of the IMV (Landzert’s fossa) [1]. The ratio of left paraduodenal hernia to right paraduodenal hernia is approximately three to one [1]. In left paraduodenal hernia patients, the median age at presentation is 47 (range of 18–82) years and the ratio of male to female is approximately three to one [3].

The clinical diagnosis is difficult because left paraduodenal hernia is asymptomatic or causes vague symptoms, such as nausea, vomiting, and postprandial pain [4]. Multi-detector CT is most useful for the diagnosis of the left paraduodenal hernia, and shows clustered dilated small-bowel loops that pass through the dorsal side of the IMV [1]. In this case, the patient had presented with repeated abdominal pain since childhood, but was not diagnosed with left paraduodenal hernia without abdominal CT. Enhanced CT revealed that almost all of the small bowel was clustered without ischemia, while the IMV ran ventrally. We were able to make a preoperative diagnosis correctly with typical CT findings.

Surgery for left paraduodenal hernia should be considered because the lifetime risk of incarceration of left paraduodenal hernia is thought to be up to 50% [4]. There are two steps in surgical procedures for left paraduodenal hernias. The first procedure is reduction of the hernia contents and the second procedure is treatment of the hernia orifice. Reduction of the hernia contents is thought to be relatively easy because of the wide hernia orifice in most cases. On the other hand, treatment of the hernia orifice is more difficult and is associated with some complications, including the IMV injury or recurrence [2]. There are two approaches to orifice treatment: closure or wide opening of the hernia orifice [2]. As IMV might be sacrificed with the wide opening approach, closure seems to be selected unless the dilatation of the small bowel is too severe or the adhesions are too strong to allow suturing of the hernia orifice [5].

Laparoscopic surgery is reported to be a useful minimally invasive technique for both the definitive diagnosis and treatment of acute small bowel obstruction and its adaptations have spread [6]. The first case of left paraduodenal hernia repaired by an entirely laparoscopic procedure was reported in 1998 [7]. Thereafter, 32 cases of left paraduodenal hernia treated by multiport laparoscopic surgery have been reported; the orifice was closed in 25 cases and orifice was opened wide in 5 cases [2]. However, there are no reports of cases treated with SILS.

SILS has the benefit of good cosmesis and the safety is assured by the easy conversion to conventional multiport laparoscopic surgery by additional ports [8]. Some reports have shown the feasibility of using SILS for other internal hernias, such as broad ligament hernia [9]. Internal hernias are sometimes difficult to be preoperatively definitively diagnosed and SILS is especially useful for internal hernia as minimally invasive surgery [9]. The needlescopic surgery is also a choice as another type of reduced port surgery [10]. However, we thought that needlescopic surgery has some drawbacks in this case. Needlescopic surgery requires specialized forceps with its small jaws and supple fine shafts that could cause the excessive tension to the protruded small bowel during pulling out through the hernia orifice [11]. Another drawback is the limitation of available devices. We cannot use laparoscopic coagulating shears (LCS) or clip appliers through the slim ports even when sudden bleeding [12]. In this case, reduction of hernia contents and treatment of the hernia orifice was necessary and preparation of LCS would have been desirable for the sudden bleeding because the hernia orifice was near the IMV. Judging comprehensively, we selected SILS in this case. However, SILS also has some drawbacks, including less flexibility of movement with interference compared to conventional multiport laparoscopic surgery including needlescopic surgery [8, 9]. We described the lists of the possible advantages and disadvantages of performing paraduodenal hernia surgery by SILS compared to multiport laparoscopic surgery (Table 1). Previous study described that SILS approach could be of interest in highly selected patients who meet the following criteria: young, mainly women, and not obese (BMI < 30) [13]. In the present case of paraduodenal hernia, we also followed these criteria. After deep consideration of these advantages and disadvantages, cases for SILS should be carefully selected.

**Table 1:** The advantages and disadvantages of performing paraduodenal hernia by single-incision laparoscopic surgery (SILS)

	SILS	Multiport laparoscopic surgery
Advantages	・Cosmetic benefits ・Possibility of less post-operative pain	・Flexibility of movement ・Some cases are previously reported
Disadvantages	・Technical difficulties ・Less counter-traction ・Interference of instruments ・Incision length can be longer	・Multiple port scars ・Incision enlargement is necessary if guiding the lesion site to the outside of the body is needed
Precautions	・Conversion to multiport laparoscopic or open surgery if safety is not guaranteed ・High-risk cases of Trocar-site hernias (e.g. obesity, umbilical hernias, diabetes mellitus or smoking, etc.)	・Conversion to open surgery if safety is not guaranteed

## Conclusions

We presented the case of left paraduodenal hernia which was preoperatively diagnosed and completely treated by SILS. SILS is a feasible diagnostic and therapeutic option with cosmetic benefits for selected patients with left paraduodenal hernia.

## Data Availability

None.

## Abbreviations

SILS: Single-incision laparoscopic surgery
CT: Computed tomography
IMV: Inferior mesenteric vein
WBC: White blood cell
CRP: C-reactive protein
LCS: Laparoscopic coagulating shears
